# Variant Angina and Cannabis-Induced Myocarditis: A Rare Presentation of Myocardial Inflammation

**DOI:** 10.7759/cureus.41196

**Published:** 2023-06-30

**Authors:** Bashar Oudah, Noor Al-Ameri, Aliaa Mousa, Hassaan Arshad, Mohammad Abu-Abaa, Sandy Park

**Affiliations:** 1 Internal Medicine, Eisenhower Medical Center, Rancho Mirage, USA; 2 Internal Medicine, Capital Health Regional Medical Center, Trenton, USA; 3 Cardiology, Eisenhower Medical Center, Rancho Mirage, USA

**Keywords:** st-elevations, chest pain, variant angina, myocarditis, marijuana abuse

## Abstract

Myocarditis is a rare but serious inflammatory disease of the myocardium, often caused by viral infections. We present a unique case of myocarditis in a previously healthy 29-year-old male who developed symptoms and electrocardiography changes of variant angina following cannabis use. This case report discusses the patient's atypical presentation, diagnostic evaluation, management, and outcome.

## Introduction

Myocarditis involves cardiac myocyte inflammation, necrosis, and degeneration, commonly affecting young individuals without atherosclerotic risk factors at an estimated incidence of 10-20 cases per 100,000 [1]. Viral infections are the most frequent cause of myocarditis. Other etiologies include bacterial and protozoal infections, toxins, medications, and autoimmune conditions [2-4]. Myocarditis presents with diverse symptoms, including chest pain, dyspnea, palpitations, and electrocardiogram changes (EKG) [5].

It can occasionally mimic ST-elevation myocardial infarction (STEMI), leading to misdiagnosis. However, coronary angiography (CAG) typically reveals normal coronary arteries [6]. Usually, ST-segment elevations observed in myocarditis are persistent rather than dynamic, with only a few reported cases showing dynamic changes, suggesting coronary artery spasm [7]. Endomyocardial biopsy is the gold standard for diagnosing myocarditis, Cardiac magnetic resonance imaging (MRI) is a valuable non-invasive tool for comprehensive myocarditis evaluation [8]. Acute myocarditis generally carries a favorable prognosis [9,10]. The association between marijuana use and myocarditis, while rare and poorly understood, has important implications due to widespread cannabis consumption [11,12].

Here, we report a rare case of myocarditis presenting as vasospastic angina pectoris in the setting of marijuana abuse.

## Case presentation

A 29-year-old male presented to the emergency department (ED) with the chief complaint of chest pain. The chest pain had started a few hours prior to presentation, was intermittent, but progressively increased in intensity, and was located retrosternally with no radiation. It was accompanied by symptoms of nausea and diaphoresis. The patient had an unremarkable past medical history, however, he smoked marijuana occasionally, which had only started recently, and admitted to smoking marijuana about five hours prior to the start of symptoms. The patient denied any recent flu-like upper respiratory symptoms or recent vaccinations including the coronavirus disease 2019 (COVID-19) vaccine. Upon arrival at the ED, the patient appeared to be resting comfortably and showed no signs of distress. He denied experiencing current chest pain. The patient had a blood pressure of 130/75 mmHg, a heart rate of 60 beats per minute, a respiratory rate of 16 cycles per minute, and an oxygen saturation level of 97% on room air. Basic laboratory tests were unremarkable (refer to Table 1).

**Table 1:** Demonstrating normal laboratory values

Variable	Value	Reference Range
white blood cell count	8.1x10^3 cells/mcL	3.8 - 10.8 x10^3 cells/mcl
hemoglobin level	13.5 g/dL	12.0 - 16.0 g/dL
platelet count	210x10^3 cells/mcL	150 - 450 x10^3 cells/mcl
serum sodium level	142 mmol/L	136 - 145 mmol/L
potassium level	4.3 mmol/L	3.5 - 5.1 mmol/L
serum creatinine level	0.8 mg/dL	0.6 - 1.2 mg/dL

**Figure 1:**
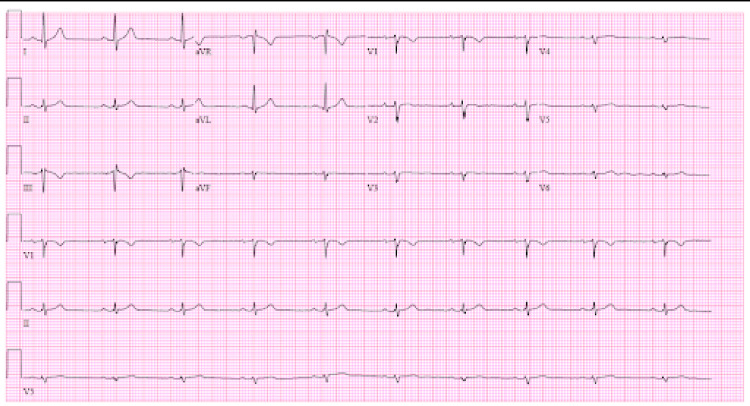
Electrocardiogram, performed as an initial evaluation showing no signs of ST-segment elevation

Serial high-sensitivity troponin I levels were elevated at 723 pg/mL (reference range <14 pg/mL) and 730 pg/mL, two hours apart, and peaked at 3350 pg/ml. The viral panel, including COVID-19, was negative. The urine drug screen was positive for cannabis only. The initial electrocardiogram (EKG) did not show any findings suggestive of acute myocardial infarction (Figure 1).

The transthoracic echocardiogram demonstrated a normal left ventricular size, wall thickness, and systolic function. The ejection fraction (EF) was measured at 60-65%, indicating normal systolic function. Additionally, the echocardiogram revealed normal diastolic function with no regional wall motion abnormalities observed. The right ventricle appeared normal in size and function. No significant valvular pathology was identified.

**Figure 2:**
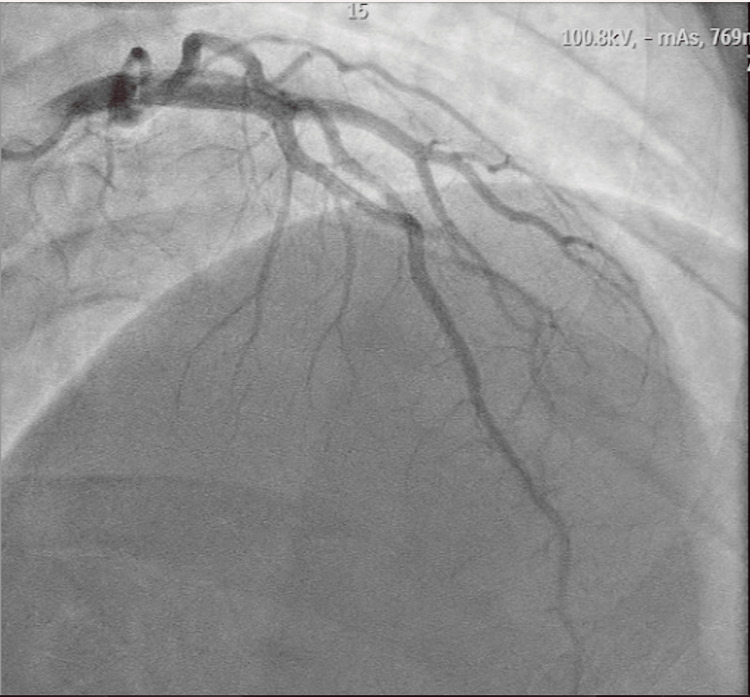
Coronary angiography in the right anterior oblique view showing a normal left coronary artery

**Figure 3:**
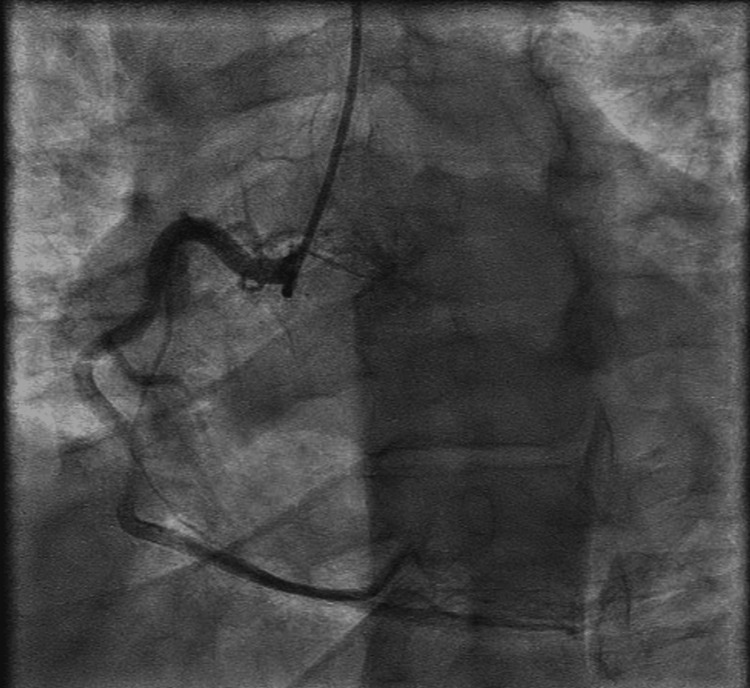
Coronary angiography in the left anterior oblique view showing the normal right coronary artery

The patient underwent coronary angiography, which revealed right-dominant coronary anatomy without any evidence of obstructive disease (Figures 2, 3).

**Figure 4:**
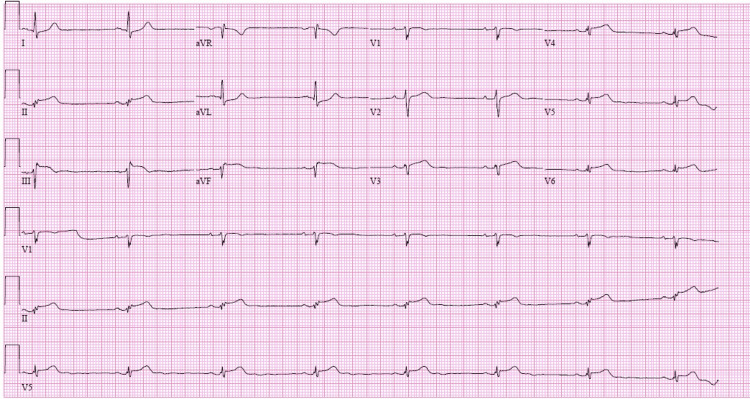
The electrocardiogram (EKG) obtained on Day 2 at 11:30 AM, during a period of chest pain It reveals ST-elevation in leads II and III and an arteriovenous fistula (AVF), suggesting an inferior wall infarction.

The patient began complaining of similar left-sided chest pain, although less intense than before. An electrocardiogram was performed to evaluate the patient's condition, revealing a new finding of ST-elevation in the II and III inferior leads and an arteriovenous fistula (AVF) (Figure 4).

**Figure 5:**
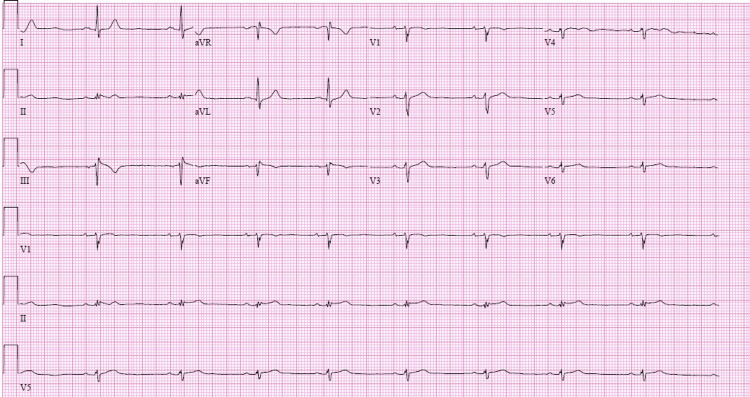
Electrocardiogram (EKG) taken on Day 2 at 12:15 PM, conducted shortly after the chest pressure subsided It demonstrates the resolution of the previously observed ST-elevation in the inferior leads.

The patient was administered sublingual nitroglycerin, which led to the complete resolution of his pain. A repeat electrocardiogram was performed (Figure 5), demonstrating the resolution of previously observed ST-elevation. A diagnosis of coronary artery spasm was made.

**Figure 6:**
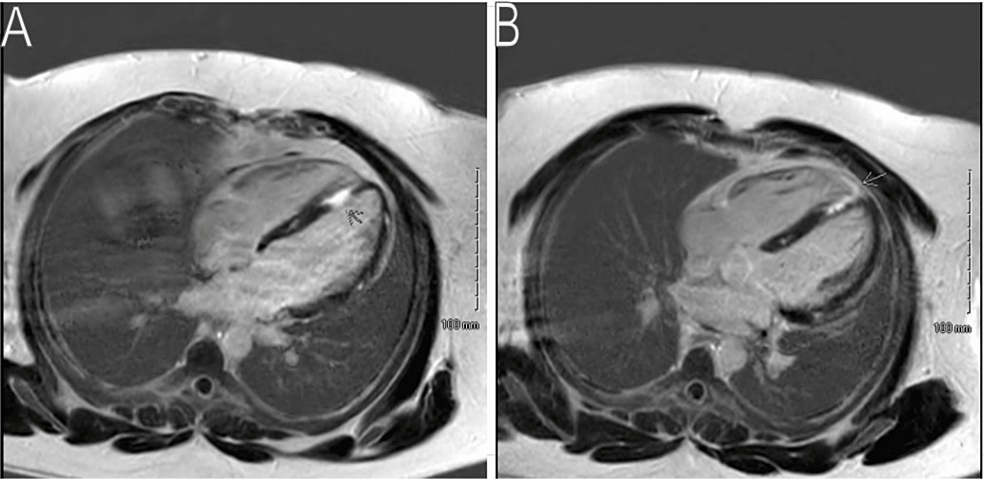
Cardiac magnetic resonance imaging findings (A and B), which demonstrate subepicardial enhancement suggestive of myocarditis (arrows)

Cardiac magnetic resonance imaging (MRI) revealed subepicardial enhancement in the septum, inferior wall, and inferolateral wall, indicating myocarditis (Figure 6). The observed pattern was atypical for infarction and suggestive of myocarditis. Additionally, diffusely increased T1 and T2 times were indicative of myocardial edema, a common finding in myocarditis. Assessment of global left ventricular (LV) systolic function showed a preserved LV ejection fraction (LVEF) of 76% and mild septal hypertrophy. The global right ventricular (RV) systolic function was also preserved, with an RV ejection fraction (RVEF) of 65%.

The patient received treatment with diltiazem and sublingual nitroglycerin as needed. Additionally, the patient was counseled on the importance of avoiding smoking marijuana. Subsequent chest pain was not reported, and the patient was discharged from the hospital.

## Discussion

Myocarditis is characterized by inflammation, necrosis, and degeneration of cardiac myocytes [1]. It is commonly observed in young individuals without atherosclerotic risk factors [1]. The estimated incidence of myocarditis is 10-20 cases per 100,000 individuals [1].

Viral infections are the most frequent cause of myocarditis, Other etiologies include bacterial and protozoal infections, toxins, medications, autoimmune conditions such as lupus, sarcoidosis, lymphocytic and giant cell myocarditis, and malignancy [2-4]. The clinical presentation of myocarditis varies widely and can range from chest pain, dyspnea, and palpitations with associated electrocardiographic (EKG) changes, to even cardiogenic shock [5].

In a comprehensive systematic review involving 1676 patients presenting with suspected myocardial infarction and non-obstructive coronary arteries, myocarditis was identified in approximately one-third (33%) of these cases [6]. Typically, ST-segment elevations observed in myocarditis are persistent rather than dynamic. This phenomenon is commonly linked to concurrent pericardial inflammation or direct inflammatory reactions within the myocardium [7]. However, a few reported cases have shown dynamic changes in the ST segment, suggesting the involvement of coronary artery spasm as a cause of chest pain in myocarditis [7].

The occurrence of coronary artery spasms in myocarditis could be attributed to the release of vasoactive substances such as thromboxane A2. These substances are produced as a consequence of platelet aggregation resulting from coronary arteritis, leading to the spasm of coronary arteries [8]. Although endomyocardial biopsy is considered the gold standard for diagnosing myocarditis, its clinical utility is restricted by the patchy distribution of inflammation [8]. Cardiac magnetic resonance imaging (MRI) serves as a valuable diagnostic tool in assessing myocarditis [9]. Echocardiography also plays a valuable role in ruling out alternative causes of heart failure and detecting the presence of ventricular thrombi. However, it does not provide specific echocardiographic findings for the diagnosis of myocarditis [10]. Cardiac MRI is the primary noninvasive imaging modality for diagnosing and evaluating myocarditis, providing evidence of myocardial inflammation, assessing fibrosis, and detecting pericardial involvement [11]. Marijuana affects the cardiovascular system by causing abnormalities in heart rate and rhythm, changes in blood pressure, vasospasm, and altered coronary blood flow [11]. Marijuana acts on CB1 and CB2 receptors. CB1 agonism is linked to atherosclerosis while CB1 antagonism and CB2 agonism have anti-atherogenic effects [12]. Contamination of marijuana with fungi, bacteria, toxins, heavy metals, and pesticides is a concern for adverse effects [12]. Although rare, there have been few reported cases linking marijuana to myocarditis [12]. The exact cause is unclear, but contaminants are considered a possible factor [12]. Further research is required to explore the relationship between marijuana use and myocardial inflammation [12].

It is essential to emphasize the significance of abstaining from marijuana use and avoiding passive smoking to prevent the occurrence and recurrence of marijuana-related myocarditis [12]. Acute myocarditis generally carries a favorable prognosis; however, it is important to note that approximately 30% of cases may progress to develop dilated cardiomyopathy [9]. Treatment focuses on managing complications and supporting cardiac function [9].

## Conclusions

In conclusion, we report a rare case of myocarditis presenting as vasospastic angina pectoris in the setting of marijuana abuse. Clinicians should maintain a high index of suspicion for myocarditis in patients with presumed myocardial infarction and normal coronary arteries. Based on reviewing previous established medical literature, we suggest that marijuana is the etiology of myocarditis in this case; this is supported by the lack of evidence of any other etiology.
